# 

**Published:** 1896-03-07

**Authors:** 


**Notice to Advertisers and Subscribers.**

All Advertisements, Orders for Copies of The Hospital and Business Communications should be addressed to **THE MANAGER, (Not to The Editor),** The Hospital, 428, Strand, London, W.C.

The Cost of Subscription, post free, is as follows:—Per week, 2½d.; per quarter, 2s. 8d.; per half-year, 5s. 3d.; per annum, 10s. 6d Foreign:—Per Annum 15s. 2d.; payable, in all cases, in advance.

### THE STUDENT OF MEDICINE.

### VACANCIES.

**Resident Medical Officers.**

The following vacancies are announced this week, the amount of salary in each case being given after the name of the institution:—

Birmingham and Midland Eye Hospital.—Assistant House Surgeon. Salary £80 per annum with apartments and board. Applications to the Medical Board by March 12th.

Boscombe Hospital, Shelley Road, Boscombe, Bournemouth.—House Surgeon; unmarried and registered. Salary £60, with board (exclusive of wine or beer), lodging, and washing. Applications to the Honorary Secretary by March 9th.

City of London Hospital for Diseases of the Chest, Victoria Park, E.—House Physician. Appointment for six months. Salary at the rate of £30 per annum, with board and residence. Applications to the Secretary by March 12th.

Northumberland County Lunatic Asylum, Morpeth.—Assistant Medical Officer; unmarried. Salary £120 per annum, increasing £10 yearly to £150, with furnished apartments, board and lodging. Applications and testimonials, endorsed "Medical," to Dr. McDowell, at the Asylum, by March 12th.

### BLACKIE AND SON'S ELEMENTARY TEXT-BOOKS.

*FULLY ILLUSTRATED.*

**ELEMENTARY PHYSIOLOGY.** By Professor AINSWORTH DAVIS. With Appendix for Agricultural Students. Cloth, 2s.

"Written in a clear, forcible style, and crammed with detailed information which is arranged with great method. The illustrations are profuse and admirable, and the directions given to students on practical work are excellent."—*British Med. Journal.*

**AN ELEMENTARY TEXT-BOOK OF PHYSIOLOGY.** By J. M'GREGOR ROBERTSON, M.A., M.B., Lecturer in Physiology, Queen Margaret College. New and Revised Edition. Cloth, 4s.

"A good system of arrangement and clear expressive exposition distinguish this book. Definitions of terms are remarkably lucid and exact."—*Saturday Review.*

**ELEMENTARY PHYSIOLOGY.** By VINCENT T. MURCHE. With Coloured Illustrations. Cloth, 2s.

"Its teaching is sound, concise, and up to date, and the care with which the little book is illustrated leaves little to be desired either by teacher or pupil."—*Hospital Gazette.*

**AN ELEMENTARY TEXT-BOOK OF ANATOMY.** By HENRY EDWARD CLARK, Fellow of the Faculty of Physicians and Surgeons of Glasgow. Cloth, 5s.

"The author's experience as a teacher and his reputation as an anatomist are a sufficient guarantee for the general accuracy of its contents. It is methodically arranged, clearly written, and well illustrated."—*Edinburgh Medical Journal.*

**ELEMENTARY HYGIENE.** By H. ROWLAND WAKEFIELD. Cloth, 2s.

"Contains a large amount of information, conveyed in clear and precise terms."—*British Medical Journal.*

London: BLACKIE and SON (Limited), Old Bailey.

Just Published. Price 2s. Net.

### STRAY THOUGHTS FOR INVALIDS,

**Original and Selected.**

By LUCY H. M. SOULSBY, Author of "Stray Thoughts for Girls."

London: Longmans, Green, and Co.

### THE 2/6 SERIES OF NURSING MANUALS.

**HOW TO BECOME A NURSE: AND HOW TO SUCCEED.** A complete guide to the nursing profession for those who wish to become Nurses, and a useful book of Reference for Nurses who have completed their training and seek employment. Compiled by HONNOR MORTEN, Author of "The Nurses' Dictionary," &c. Third and Revised Edition. Demy 8vo, 209 pp., profusely Illustrated with Copyright Portraits and Drawings.

"To those who are frequently appealed to by girls, or young women, as to the steps they should take to become nurses, this book of Miss Morten's must prove a perfect godsend."—*British Medical Journal.*

**THE NURSES' DICTIONARY OF MEDICAL TERMS AND NURSING TREATMENT.** Compiled for the use of Nurses, by HONNOR MORTEN. Containing descriptions of the principal Medical and Nursing Terms and Abbreviations, Instruments, Drugs, Diseases, Accidents, Treatments, Physiological Names, Operations, Foods, Appliances, &c., &c., encountered in the Ward or Sick-room. Third and Revised Edition (25th thousand). Demy 16mo, suitable for the apron pocket, handsomely bound in handsome leather, gilt, price 2s. 6d. net, or in cloth, price 2s.

"Should be at the disposal of every nurse."—*Birmingham Medical Review.*

**HOSPITAL SISTERS AND THEIR DUTIES.** By EVA C. LUCKES, Matron to the London Hospital. The favourable reception accorded to the first two editions of this useful and interesting work, and the regular demand for copies of it, encourage the publishers to place this third edition, greatly improved and enlarged, before the Institutions. The experience and position of its author entitle it to authority, and every Matron, Sister, or Nurse who aspires to become a Sister, should read the advice and information contained in its pages. Third Edition. Enlarged and partly rewritten, crown 8vo, cloth gilt, about 200 pp.

**MENTAL NURSING.** A Text-book specially designed for the instruction of Attendants on the Insane. By WILLIAM HARDING, M.D., M.R.C.P. The want of a complete book for the instruction of Asylum Attendants has been long felt, and it is with confidence that the publishers recommend this work to the managers of all Institutions for the Insane. Second Edition. Enlarged, demy 8vo, profusely illustrated, nearly 200 pp., cloth.

**SPINAL CURVATURE AND AWKWARD DEPORTMENT: Their Causes and Prevention in Children.** By Dr. GEORGE MULLER, Professor of Medicine and Orthopædics, Berlin. English Edition, edited and adapted by RICHARD GREENE, F.R.C.P. Crown 8vo, cloth gilt, with many Plates and Illustrations.

"Dr. Müller's little book is an able essay upon the effect produced by exercise, attitude, and movement upon the growth and development of the body. He further gives directions which any intelligent parent or teacher could carry out with the help of very simple apparatus, showing how to avoid or, in early cases, to cure certain malformations which, in the majority of cases, arise either from the neglect of very simple hygienic rules or from carelessness."—*Daily Chronicle.*

**THE MOTHER'S HELP AND GUIDE TO THE DOMESTIC MANAGEMENT OF CHILDREN.** By P. MURRAY BRAIDWOOD, M.D., formerly Senior Medical Officer to the Wirral Hospital for Sick Children. Crown 8vo, cloth gilt.

"The book is altogether admirable."—*Manchester Courier.*

London: THE SCIENTIFIC PRESS (Limited), 428, STRAND, W.C.

*In the Press. Price 5s.*

### BURDETT'S OFFICIAL NURSING DIRECTORY

A DIRECTORY, NOT A REGISTER.

THE HOSPITAL states: "It is well to bear in mind the difference and distinction which exists between a register and a directory. A register implies rights, and suggests that those who are not upon it are in some way or another inferior beings. But a directory is another matter. A directory gives as rights; insertion in it implies no adherence to any particular party or any particular opinion, and in no way interferes with entry on the register of any association, or the list of any institute, to which the individual may belong. All that a directory professes to do is to give information. The 'Medical Register' is not a mere list of qualified medical men; it is admission to the Register, which itself makes him qualified. For years, perhaps even now, 'Churchill's Medical Directory' contained the names of medical men who, so far as public appointments were concerned, were unqualified; it also contains the names of homœopathists, whom four out of every five medical men will not meet; and it certainly contains the names of many for whose moral character we should not like to vouch. But it gives full and accurate information about them all, and for the sake of that information it is constantly consulted both by the public and the doctors."

**The above work will be ready early in the Spring, and those Nurses who have not already sent in their names should at once apply for a form, which can be obtained upon application to the Publishers,**

THE SCIENTIFIC PRESS (Lim.), 428, Strand, London, W.C.

